# AI anxiety and AI learning intention among Chinese university students: a protection motivation theory perspective

**DOI:** 10.3389/fpsyg.2026.1865077

**Published:** 2026-07-10

**Authors:** Qin Wu, Wang He

**Affiliations:** 1 College of Humanities and Law, Shanghai Business School, Shanghai, China; 2 School of International Economics and Politics, Jiangxi University of Finance and Economics, Nanchang, China

**Keywords:** AI anxiety, AI learning intention, Chinese university students, fsQCA, protection motivation theory, response efficacy, self-efficacy

## Abstract

The rapid development of artificial intelligence (AI) has introduced new psychological and educational challenges for university students. In higher education settings, students may perceive AI as a source of employment pressure, skill renewal demands, and future uncertainty, while also regarding AI learning as an important form of academic and career preparation. Drawing on Protection Motivation Theory (PMT), this study examines the associations among perceived threat severity, perceived threat vulnerability, response efficacy, self-efficacy, AI anxiety, protection motivation, and AI learning intention among Chinese university students. Based on cross-sectional survey data from 350 undergraduates in mainland China, structural equation modeling (SEM) was used to test the proposed relationships, and fuzzy-set qualitative comparative analysis (fsQCA) was further used to identify configurations associated with high AI learning intention. The SEM results indicated good model fit, χ^2^/df = 1.629, RMSEA = 0.042, TLI = 0.948, CFI = 0.952, and IFI = 0.952. Perceived threat severity and perceived threat vulnerability were positively associated with both AI anxiety and protection motivation. Self-efficacy and AI anxiety were positively associated with protection motivation, whereas response efficacy showed a positive but statistically non-significant direct association with protection motivation. Protection motivation was positively associated with AI learning intention. The mediation results suggested small but statistically significant indirect associations between threat perceptions and protection motivation through AI anxiety. The fsQCA results complemented the SEM findings by identifying multiple configurations associated with high AI learning intention, including an efficacy-motivation configuration, a threat-motivation configuration, and an anxiety-efficacy configuration. These findings suggest that AI learning intention among Chinese university students is associated with the joint presence of threat appraisal, efficacy beliefs, AI anxiety, and protection motivation rather than any single psychological factor. This study contributes incremental evidence to AI learning research by clarifying how cognitive, emotional, and motivational factors are related within a PMT-based framework.

## 1 Introduction

Artificial intelligence (AI) is increasingly embedded in higher education, employment preparation, and everyday learning practices. For university students, AI is no longer only a technological topic discussed in abstract terms. It is becoming closely related to coursework, information search, writing support, programming assistance, skill development, and future career planning. With the rapid diffusion of generative AI and AI-enabled learning tools, students are increasingly exposed to questions about technological substitution, changing skill requirements, and competition in the labor market. As a result, their responses to AI may involve not only perceived usefulness or ease of use, but also worry, uncertainty, and pressure concerning future academic and occupational adaptation.

This issue is particularly salient in Chinese higher education. Chinese university students are situated in an educational environment characterized by strong performance expectations, intensive credential competition, and increasing pressure to improve employability. In this context, AI is often perceived not only as a useful learning tool, but also as an external force that may reshape the value of existing knowledge and the criteria of future career competitiveness. Students may therefore evaluate AI in relation to personally relevant concerns: whether their current skills will remain valuable, whether their major or intended occupation will be affected, and whether they can acquire the AI-related competencies required for future opportunities. These conditions make Chinese higher education an important context for examining how AI-related threat appraisal, anxiety, efficacy beliefs, and learning intention are associated.

Existing research has provided important explanations for students’ technology-related behavior. Technology acceptance models and related frameworks have emphasized cognitive evaluations such as perceived usefulness, perceived ease of use, attitudes, facilitating conditions, and social influence. These perspectives have substantially advanced understanding of why learners adopt or use digital technologies. However, they are less well suited to explaining why students may express willingness to learn AI even when they experience anxiety or perceive AI as threatening. In AI-related educational settings, students’ learning intention may not arise only from positive evaluations of usefulness. It may also be associated with perceived risks, employment pressure, emotional unease, and the belief that learning AI is necessary for future adaptation.

Recent studies on AI anxiety have begun to address this issue. AI anxiety generally refers to feelings of worry, tension, uncertainty, or fear arising from AI technologies and their perceived consequences. In educational contexts, AI anxiety may involve concerns about learning difficulty, job replacement, professional devaluation, overreliance on AI, and uncertainty in human-AI interaction. Prior research has shown that AI anxiety can be negatively related to technology acceptance or learning engagement. At the same time, emerging studies suggest that AI anxiety may coexist with learning motivation under certain conditions, especially when learners report stronger AI self-efficacy or clearer beliefs about the value of AI learning. Cheng (2025), Chen et al. (2025), and Teke (2025), for example, have provided recent evidence that AI-related anxiety, efficacy beliefs, and learning motivation are closely connected. These studies indicate that AI anxiety should not be treated only as a barrier variable. Instead, its role needs to be examined in relation to threat appraisal, coping appraisal, and motivation.

Protection Motivation Theory (PMT) provides a useful framework for this purpose. PMT proposes that individuals’ protective motivation is associated with two broad forms of appraisal: threat appraisal and coping appraisal. Threat appraisal includes perceived severity and perceived vulnerability, whereas coping appraisal includes response efficacy and self-efficacy. In traditional PMT applications, these constructs have often been used to explain health protection, cybersecurity behavior, or other risk-related actions. In AI learning contexts, however, the perceived threat is not usually physical harm. It is more often related to skill obsolescence, employment competition, learning gaps, and future uncertainty. Similarly, the protective response is not avoidance of a harmful object, but an adaptive learning orientation toward acquiring AI-related knowledge and skills.

Applying PMT to AI learning intention therefore requires some conceptual adjustment. Perceived threat severity refers to students’ judgments about the seriousness of AI-related consequences for future learning and employment. Perceived threat vulnerability refers to the extent to which students believe that they personally may be affected by AI-related changes. Response efficacy refers to the belief that learning AI can help address these challenges, whereas self-efficacy refers to students’ confidence in their ability to learn and use AI-related knowledge or tools. AI anxiety captures the emotional response associated with perceived technological pressure. Protection motivation, in this study, refers to students’ motivational readiness to improve AI-related competencies in response to perceived uncertainty and future demands. AI learning intention refers to their stated willingness to continue learning AI knowledge, tools, and applications.

Although PMT has recently been applied to technology and AI-related contexts, three gaps remain. First, existing AI learning research has paid more attention to use intention than to learning intention. The intention to use AI tools and the intention to learn AI are conceptually related but not identical. Tool use often reflects immediate instrumental behavior, whereas AI learning intention reflects a broader willingness to invest time and effort in developing future-oriented competencies. Second, prior studies have not sufficiently integrated threat cognition, emotional response, and efficacy beliefs into one explanatory framework. AI anxiety is often examined as an outcome, a barrier, or a moderator, but its role in the association between threat appraisal and protection motivation remains less fully specified. Third, although the combination of SEM and fsQCA has become increasingly common in education and information systems research, its value depends on whether it offers insights beyond average net effects. In the present study, SEM is used to examine the overall associations among constructs, whereas fsQCA is used to identify different configurations of conditions associated with high AI learning intention.

The present study therefore examines AI learning intention among Chinese university students through a PMT-based framework integrating threat appraisal, AI anxiety, efficacy beliefs, and protection motivation. Importantly, because the study is based on cross-sectional self-reported survey data, it does not claim to demonstrate causal processes or temporal changes. Instead, the analysis focuses on structural associations, indirect relationships, and configurational patterns. This more cautious framing is consistent with the empirical design and helps avoid overinterpreting mediation results as evidence of psychological change over time.

This study makes three incremental contributions. First, it extends PMT to the context of AI learning intention by distinguishing learning AI from merely using AI tools. This allows the study to focus on students’ willingness to develop AI-related competencies rather than their immediate acceptance of specific technologies. Second, it places AI anxiety within a PMT-based framework as an emotional construct associated with threat appraisal and protection motivation. This approach contributes to the emerging literature on AI anxiety by examining its role alongside perceived severity, perceived vulnerability, response efficacy, and self-efficacy. Third, by combining SEM and fsQCA, the study distinguishes between average structural associations and multiple configurations linked to high AI learning intention. This is especially useful for interpreting response efficacy: although it may not show a significant direct association in the SEM model, it may still appear as an important condition within specific configurations. In this way, the study provides a more nuanced account of how cognitive, emotional, and motivational conditions are jointly related to AI learning intention in Chinese higher education.

Based on these considerations, the study addresses the following research questions: How are perceived threat severity, perceived threat vulnerability, response efficacy, self-efficacy, AI anxiety, and protection motivation associated with AI learning intention among Chinese university students? Does AI anxiety show an indirect association between threat appraisal and protection motivation? Does protection motivation link threat appraisal, efficacy beliefs, and AI anxiety with AI learning intention? What configurations of threat appraisal, efficacy beliefs, AI anxiety, and protection motivation are associated with high AI learning intention?

## 2 Theoretical background and hypotheses

### 2.1 Protection motivation theory and AI learning intention

Protection Motivation Theory provides a useful framework for explaining how individuals respond to perceived risks and uncertainty. According to PMT, individuals’ motivational responses are associated with two broad appraisal processes: threat appraisal and coping appraisal. Threat appraisal usually includes perceived severity and perceived vulnerability, whereas coping appraisal includes response efficacy and self-efficacy. In traditional applications, PMT has often been used to examine health-related protection, cybersecurity behavior, and other forms of risk-avoidance or risk-reduction behavior. In these contexts, protection motivation refers to individuals’ willingness to adopt behaviors that reduce perceived harm or vulnerability.

The AI learning context differs from many traditional PMT settings. For university students, AI-related threats are not primarily physical or immediate. They are more often associated with perceived skill obsolescence, employment competition, changing knowledge requirements, and uncertainty about future academic and occupational adaptation. Similarly, the relevant protective response is not avoidance of AI, but a willingness to learn AI-related knowledge and skills. AI learning intention therefore represents a capability-oriented form of adaptive motivation. It reflects students’ stated willingness to invest time and effort in understanding AI, practicing AI tools, and improving their preparedness for future learning and employment environments.

Recent studies have begun to apply PMT to AI-related learning and technology-use contexts. Cheng (2025), for example, examined university students’ intention to learn AI by integrating expectancy-value theory and PMT. This work is particularly important because it has already shown that PMT can be meaningfully extended to AI learning intention. The present study therefore does not claim to be the first to apply PMT to AI learning. Instead, it builds on this emerging line of work by further incorporating AI anxiety as an emotional construct within a PMT-based framework and by examining how threat appraisal, efficacy beliefs, anxiety, and protection motivation are jointly associated with AI learning intention.

This distinction is important. Much existing AI education research focuses on whether students accept or use AI tools, while AI learning intention refers to a broader willingness to develop AI-related competencies. The latter is especially relevant in higher education because students are not only users of AI applications; they are also future workers who may need to understand, evaluate, and adapt to AI-mediated environments. Thus, extending PMT to AI learning intention helps shift the analytical focus from short-term tool acceptance to longer-term capability preparation. However, PMT alone may not fully explain students’ responses to AI because it emphasizes cognitive appraisal more than emotional experience. This creates a need to examine AI anxiety more explicitly within the PMT framework.

### 2.2 AI anxiety in learning and technology-adoption contexts

AI anxiety refers to feelings of worry, tension, uncertainty, or unease that individuals experience when facing AI technologies and their possible consequences. In educational contexts, AI anxiety may involve concerns about learning difficulty, insufficient AI knowledge, job replacement, professional devaluation, overreliance on AI, and uncertainty in human-AI interaction. Compared with general technology anxiety, AI anxiety is more closely related to students’ perceived future competitiveness and career adaptation because AI is often understood as a technology that can change both learning practices and occupational structures.

Previous research has often treated anxiety as a barrier to technology acceptance or learning engagement. From this perspective, anxiety may reduce students’ confidence, increase avoidance, and weaken their intention to use unfamiliar technologies. This interpretation remains important in AI learning contexts because some students may avoid AI-related learning when they perceive AI as too difficult, too uncertain, or too threatening. However, recent studies suggest that the role of AI anxiety may be more complex. Under certain conditions, AI anxiety may coexist with stronger learning motivation or greater willingness to improve AI-related skills.

Chen et al. (2025) provided important evidence in this direction by examining the relationship between AI anxiety, AI self-efficacy, and motivated learning among undergraduate students. Their study suggests that AI anxiety is not necessarily only an inhibiting factor; its association with motivated learning may depend on learners’ self-efficacy. Teke (2025) reached a related conclusion in the context of pre-service teachers, suggesting that AI anxiety may be associated with motivated learning when it is accompanied by sufficient AI self-efficacy. These studies are directly relevant to the present research because they show that AI anxiety may be connected to learning motivation in a more conditional way than traditional anxiety-based explanations assume.

At the same time, the present study differs from these studies in three respects. First, rather than focusing primarily on the direct association between AI anxiety and motivated learning, this study places AI anxiety within a PMT-based structure of threat appraisal, coping appraisal, protection motivation, and AI learning intention. Second, rather than examining self-efficacy alone as the key boundary condition, this study considers both self-efficacy and response efficacy, thereby distinguishing students’ perceived capability to learn AI from their belief that AI learning is useful for coping with future challenges. Third, this study uses SEM and fsQCA in a complementary way. SEM is used to examine average structural associations, whereas fsQCA is used to identify configurations in which AI anxiety may appear together with efficacy beliefs and protection motivation.

Therefore, this study does not argue that AI anxiety is inherently beneficial or that anxiety is transformed into learning intention. Such claims would exceed what can be supported by cross-sectional survey data. Instead, the study treats AI anxiety as an emotional construct that may be positively or negatively related to learning motivation depending on the broader configuration of threat appraisal and efficacy beliefs. This more cautious view is consistent with recent AI anxiety research while allowing the present study to clarify the specific role of AI anxiety within PMT.

### 2.3 Self-efficacy and response efficacy in AI learning

Wang and Chuang (2024) developed and validated an artificial intelligence self-efficacy scale, providing a measurement basis for subsequent AI learning research. Coping appraisal is a central component of PMT. It involves individuals’ judgments about whether a protective response is effective and whether they are capable of performing that response. In the context of AI learning, response efficacy refers to students’ belief that learning AI can help them cope with future learning, employment, and skill-renewal pressures. Self-efficacy refers to students’ belief that they are capable of understanding AI-related knowledge, using AI tools, and solving problems encountered in AI learning.

The distinction between response efficacy and self-efficacy is theoretically important. A student may believe that AI learning is valuable but still feel unable to learn AI effectively. Conversely, a student may feel capable of learning AI but doubt whether AI learning will meaningfully improve future competitiveness. PMT suggests that both beliefs may be associated with protection motivation, but their roles may differ across contexts. In AI education, self-efficacy may be especially close to students’ willingness to engage in learning because AI is often perceived as technically complex and rapidly changing. When students believe that they can learn AI, they may be more likely to form motivation to improve their AI-related competencies.

Recent AI learning studies also highlight the importance of efficacy beliefs. Chen et al. (2025) emphasized that AI self-efficacy shapes the relationship between AI anxiety and motivated learning among undergraduates. Teke (2025) similarly showed that AI self-efficacy is important for understanding when AI anxiety is associated with motivated learning among pre-service teachers. These findings suggest that efficacy beliefs provide a key psychological condition under which AI-related anxiety is linked to adaptive learning responses.

However, response efficacy has received less attention than self-efficacy in much of the AI anxiety literature. This is a limitation because students’ AI learning intention may depend not only on whether they believe they can learn AI, but also on whether they believe AI learning is a meaningful response to future uncertainty. Cheng (2025) has already demonstrated the relevance of PMT to AI learning intention, but further work is needed to clarify the differentiated roles of response efficacy and self-efficacy when AI anxiety is also included in the model. This study therefore examines both efficacy constructs simultaneously and treats them as complementary rather than interchangeable dimensions of coping appraisal.

### 2.4 Integrating PMT, AI anxiety, and configurational thinking

Taken together, the existing literature suggests that AI learning intention is associated with cognitive, emotional, and motivational factors. PMT explains how perceived threats and coping beliefs are related to protection motivation. AI anxiety research shows that students’ emotional responses to AI may be linked to learning motivation in conditional ways. Efficacy-based studies further indicate that students’ confidence in their ability to learn AI and their belief in the usefulness of AI learning may shape how they respond to AI-related uncertainty. These perspectives are complementary, but they have not always been integrated within one framework.

The present study therefore develops a PMT-based framework in which perceived threat severity and perceived threat vulnerability represent threat appraisal; response efficacy and self-efficacy represent coping appraisal; AI anxiety represents an emotional response associated with perceived AI-related uncertainty; protection motivation represents students’ motivational readiness to improve AI-related competencies; and AI learning intention represents their stated willingness to continue learning AI knowledge, tools, and applications. In this framework, AI anxiety is not assumed to be a uniformly negative barrier or a uniformly positive driver. Rather, it is examined as an emotional construct associated with threat appraisal and protection motivation.

This framework helps clarify the incremental contribution of the present study relative to recent work. Compared with Cheng (2025), the study gives greater attention to AI anxiety and examines whether it has an indirect association between threat appraisal and protection motivation. Compared with Chen et al. (2025) and Teke (2025), the study embeds AI anxiety within PMT rather than examining it mainly in relation to self-efficacy and motivated learning. In addition, the study uses fsQCA to examine whether different combinations of threat appraisal, efficacy beliefs, AI anxiety, and protection motivation are associated with high AI learning intention. This is useful because a non-significant average association in SEM does not necessarily mean that a condition is irrelevant in all cases. A condition may be important only when it appears together with other conditions.

This configurational perspective is especially relevant for response efficacy. In a traditional SEM model, response efficacy may show a weak or non-significant direct association with protection motivation. However, from a configurational perspective, response efficacy may still be associated with high AI learning intention when it is combined with high self-efficacy, AI anxiety, or protection motivation. Thus, fsQCA does not serve as a methodological novelty in itself. Its value lies in showing whether multiple pathways are associated with high AI learning intention and whether some constructs operate as conditional contributors rather than as strong net-effect predictors.

Based on the above discussion, this study addresses three research gaps. First, although PMT has begun to be applied to AI learning intention, further evidence is needed on how AI anxiety can be incorporated into a PMT-based model without treating it as either purely harmful or inherently beneficial. Second, although recent studies have examined the facilitating or conditional role of AI anxiety, less is known about how AI anxiety is associated with threat appraisal and protection motivation within a broader risk-efficacy framework. Third, existing SEM-based studies provide important evidence on average relationships among variables, but they provide less insight into alternative configurations of psychological conditions associated with high AI learning intention. The present study responds to these gaps by combining SEM and fsQCA to examine both structural associations and configurational patterns among Chinese university students.

## 3 Hypotheses development

### 3.1 Perceived threat

Perceived threat is a core antecedent in Protection Motivation Theory and is usually composed of perceived threat severity and perceived threat vulnerability (Fatimah, 2022; Reed et al., 2023). Perceived threat severity emphasizes individuals’ judgment of how serious potential consequences may be, whereas perceived threat vulnerability reflects their perceived likelihood of personally encountering relevant risks.

In the present study, perceived threat severity refers mainly to university students’ judgments about the seriousness of AI-related employment, competence-related, and social-adaptation risks. Perceived threat vulnerability refers to students’ judgments about the likelihood that they themselves may be affected by these risks. Prior PMT research suggests that higher levels of threat perception may be associated not only with stronger emotional responses, such as anxiety, but also with stronger motivation to adopt protective or adaptive actions. Based on this reasoning, the following hypotheses are proposed:

*H1*: Perceived threat severity is positively associated with protection motivation.

*H2*: Perceived threat severity is positively associated with AI anxiety.

*H3*: Perceived threat vulnerability is positively associated with AI anxiety.

*H4*: Perceived threat vulnerability is positively associated with protection motivation.

### 3.2 Perceived efficacy: a dual perspective on self-efficacy and response efficacy

At the theoretical level, self-efficacy emphasizes individuals’ judgment of whether they can successfully complete a given task (Bandura, 1997), whereas response efficacy emphasizes their judgment of whether a given behavior can effectively reduce a relevant risk. From the perceived-efficacy perspective, these two constructs are not substitutes for one another; rather, they jointly constitute important components of coping appraisal when individuals face potential threats. Recent experimental evidence also indicates that self-efficacy and response efficacy are integrated in decision-making processes and jointly shape action choices (Yang and Delgado, 2025).

In AI learning settings, response efficacy can be understood as university students’ judgment of whether learning AI can help reduce future risks and enhance competitiveness, whereas self-efficacy refers to their judgment of whether they are capable of mastering AI-related knowledge and skills. Recent studies indicate that AI self-efficacy is associated not only with AI use and learning engagement, but also with emotional adjustment and learning involvement in AI-supported educational environments (Chen et al., 2024; Qiu, 2025; Teng et al., 2026). Theoretically, when students believe that learning AI has practical value and also perceive themselves as capable of mastering relevant knowledge, their protection motivation should be more likely to be activated. At the same time, these effects may vary across different combinations of psychological conditions. Based on this reasoning, the following hypotheses are proposed:

*H5*: Response efficacy is positively associated with protection motivation.

*H6*: Self-efficacy is positively associated with protection motivation.

### 3.3 AI anxiety and its mediating role

AI anxiety refers to worry, unease, or fear surrounding AI technologies and their applications. Its content may involve job replacement, uncertainty in human-AI interaction, privacy and security concerns, and broader social and ethical issues (Johnson and Verdicchio, 2017; Chen et al., 2024; Kai et al., 2026; Li and Huang, 2020; Wang and Wang, 2022).

Existing research offers two broad interpretations of AI anxiety. One line of research emphasizes its inhibiting role, arguing that anxiety may reduce learning engagement, technology-use intention, willingness to communicate, or career certainty (Chen et al., 2024; Zhang et al., 2024). Another line suggests that when individuals possess higher self-efficacy or clearer coping pathways, AI anxiety may coexist with stronger learning motivation or adaptive action tendencies (Chen et al., 2025; Teke, 2025; Duan et al., 2026; Zhang et al., 2022).

Accordingly, the present study does not treat AI anxiety as a simple unidirectional negative variable. Instead, it focuses on whether AI anxiety has an indirect association between threat perceptions and protection motivation. In other words, university students’ perceptions of AI-related threats may be associated with emotional responses, which may in turn be linked to stronger motivation to improve AI-related competencies. Because the present study is based on cross-sectional data, the hypotheses are framed as indirect associations rather than causal processes. Based on this reasoning, the following hypotheses are proposed:

*H7*: AI anxiety is positively associated with protection motivation.

### 3.4 Protection motivation and its mediating role

In PMT, protection motivation refers to the protective action intention that individuals form after completing threat appraisal and coping appraisal; that is, it represents the psychological tendency to adopt adaptive behavior in order to avoid or reduce potential harm (Rogers, 1983). In AI learning settings, such protective action can be expressed as students’ willingness to actively learn AI-related knowledge and skills so as to prepare for future uncertainty. Studies of AI service adoption, AI learning, and educational AI use also suggest that protection motivation can connect risk cognition with specific behavioral intentions (Park et al., 2024; Cheng, 2025; Yu et al., 2026).

The perceived-efficacy perspective further helps explain the formation of protection motivation. If students believe that learning AI can help them cope with future risks (response efficacy) and also believe that they are capable of completing relevant learning tasks (self-efficacy), their protection motivation is more likely to be activated. At the same time, AI anxiety may be associated with protection motivation when it is accompanied by manageable coping beliefs and a clearer sense of action direction (Chen et al., 2025; Teke, 2025; Yang and Delgado, 2025).

Accordingly, this study places protection motivation in the key transmission position of the integrated model. The hypotheses are organized sequentially to clarify two forms of indirect association. First, AI anxiety is expected to link threat appraisal with protection motivation. Second, protection motivation is expected to link threat appraisal, efficacy beliefs, and AI anxiety with AI learning intention. The specific hypotheses are as follows:

*H8*: Protection motivation is positively associated with AI learning intention.

*H9*: Perceived threat severity is indirectly associated with protection motivation through AI anxiety.

*H10*: Perceived threat vulnerability is indirectly associated with protection motivation through AI anxiety.

*H11*: Perceived threat severity is indirectly associated with AI learning intention through protection motivation.

*H12*: AI anxiety is indirectly associated with AI learning intention through protection motivation.

*H13*: Perceived threat vulnerability is indirectly associated with AI learning intention through protection motivation.

*H14*: Response efficacy is indirectly associated with AI learning intention through protection motivation.

*H15*: Self-efficacy is indirectly associated with AI learning intention through protection motivation.

**Figure 1:**
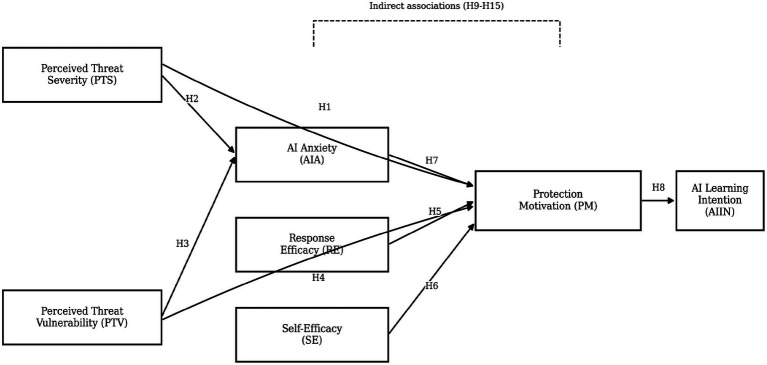
Research model of factors and mechanisms influencing AI learning intention.

Based on the above theoretical reasoning, this study develops an integrated PMT-based model while drawing on self-efficacy theory and the perceived-efficacy perspective. The model emphasizes the structural pathways through which threat appraisal, coping appraisal, AI anxiety, and protection motivation are associated with university students’ AI learning intention. Specifically, perceived threat severity and perceived threat vulnerability are expected to be positively associated with protection motivation and AI anxiety; response efficacy and self-efficacy are expected to be positively associated with protection motivation; AI anxiety is expected to be positively associated with protection motivation and to show indirect associations between threat appraisal and protection motivation; and protection motivation is expected to be positively associated with AI learning intention and to link threat appraisal, efficacy beliefs, and AI anxiety with AI learning intention. Figure 1 presents the conceptual model with H1–H15 arranged in sequential order.

## 4 Materials and methods

### 4.1 Participants and data collection

This study adopted a cross-sectional survey design to examine the associations among perceived threat severity, perceived threat vulnerability, response efficacy, self-efficacy, AI anxiety, protection motivation, and AI learning intention among Chinese university students. The target participants were undergraduate students enrolled in universities in mainland China. To ensure that the respondents were appropriate for the research purpose, the questionnaire included two screening questions before the formal survey: whether the participant was at least 18 years old and whether the participant was currently an undergraduate student in mainland China. Respondents who did not meet these criteria were not allowed to proceed to the formal questionnaire.

The questionnaire was distributed online through Wenjuanxing. Before completing the questionnaire, participants were informed of the purpose of the study, the voluntary nature of participation, the anonymity of the questionnaire, the right to withdraw, and the use of data for academic research only. A total of 352 questionnaires were collected. After excluding invalid responses based on screening criteria, completeness, and the attention-check item, 350 valid responses were retained for analysis, yielding an effective response rate of 99.43%.

The final sample included 166 male students and 184 female students, accounting for 47.4 and 52.6% of the sample, respectively. In terms of grade level, 68 were first-year students, 123 were second-year students, 95 were third-year students, and 64 were fourth-year students. In terms of academic background, 221 students were from science, engineering, agriculture, or medicine-related majors, and 129 students were from economics, management, or law-related majors. Overall, the sample showed variation in gender, grade level, and disciplinary background, providing a basis for examining AI-related psychological responses among Chinese university students (see Table 1).

**Table 1:** Sample characteristics.

Characteristic	Category	*n*	%
Gender	Male	166	47.4
Gender	Female	184	52.6
Grade	First year	68	19.4
Grade	Second year	123	35.1
Grade	Third year	95	27.1
Grade	Fourth year	64	18.3
Major	Science/engineering/agriculture/medicine	221	63.1
Major	Economics/management/law	129	36.9

### 4.2 Ethical considerations

This study received supplementary ethics review and exemption confirmation from the Academic Ethics Committee of the College of Humanities and Law, Shanghai Business School. The reference number is WFXY2026-003. The ethics committee confirmed that the study was an anonymous, non-interventional online questionnaire survey in the field of educational psychology and learning behavior. The study did not involve medical intervention, clinical treatment, biological sample collection, invasive procedures, or identifiable personal information. Therefore, it was considered a minimal-risk anonymous questionnaire study and was eligible for exemption from full ethics review or for a simplified ethics review procedure.

All participants provided informed consent before completing the questionnaire. The informed consent text explained the research purpose, anonymity, voluntary participation, right to withdraw, confidentiality measures, and data-use arrangements. Participants were informed that they could stop answering or withdraw from the survey at any time without any adverse consequences. No names, student numbers, identity card numbers, phone numbers, precise addresses, or other directly identifiable information were collected. The data were used only for academic analysis, and the research results are reported only in aggregate statistical form.

### 4.3 Measures

All formal measurement items were assessed using a seven-point Likert scale, ranging from 1 = strongly disagree to 7 = strongly agree. The questionnaire measured seven latent constructs: perceived threat severity, perceived threat vulnerability, response efficacy, self-efficacy, AI anxiety, protection motivation, and AI learning intention.

Perceived threat severity and perceived threat vulnerability were adapted from the threat-appraisal dimensions of PMT and were contextualized to the AI learning and employment environment. Response efficacy and self-efficacy were adapted from the coping-appraisal dimensions of PMT and self-efficacy research. Protection motivation measured students’ motivational readiness to improve AI-related competencies in response to perceived future uncertainty. AI learning intention measured students’ stated willingness to learn AI-related knowledge, practice AI tools, participate in AI-related courses or training, and continue following AI applications.

The 12-item AI anxiety scale was not copied from a single existing instrument. Instead, it was developed and contextualized for this study based on prior AI anxiety, technology anxiety, and AI learning research (Li and Huang, 2020; Wang and Wang, 2022; Wang et al., 2022). The item pool was designed to cover four content domains that are relevant to university students: AI learning anxiety, occupational substitution anxiety, uncertainty in human-AI interaction, and broader pressure arising from AI-related social and occupational change. These domains were used to ensure content coverage during scale development rather than to specify four separate subscales in the structural model. Accordingly, AI anxiety was modeled as a global latent construct in the main SEM analysis. The initial item pool was reviewed by experts in educational psychology and AI-related learning research. A pilot test with 20 undergraduate students was then conducted to assess item clarity, wording, and contextual relevance. Based on feedback from the pilot test and expert consultation, the wording of several items was revised before the formal questionnaire was administered. The measurement items are reported in the Supplementary material.

### 4.4 Data quality control and common method bias

Several procedural measures were adopted to improve data quality and reduce potential response bias. First, the questionnaire was anonymous and did not collect directly identifiable personal information. Second, participants were informed that there were no right or wrong answers and were asked to respond according to their true feelings. Third, the questionnaire included screening questions to ensure that respondents were adult undergraduate students in mainland China. Fourth, an attention-check item was inserted into the questionnaire. Responses that failed the attention-check requirement were treated as invalid and excluded from the final dataset. Fifth, item wording was reviewed through expert consultation and pilot testing to improve clarity and reduce ambiguity.

Because all variables were measured using self-reported questionnaire data collected at a single time point, common method bias was assessed using both procedural and statistical approaches. Harman’s single-factor test was conducted by entering all measurement items into an unrotated exploratory factor analysis. The results showed that the first factor explained 37.60% of the total variance, below the commonly used threshold of 50%, suggesting that a single factor did not account for the majority of covariance among the measurement items. In addition, a full collinearity VIF test was conducted at the construct level following Kock’s (2015) recommendation. The full collinearity VIF values ranged from 1.474 to 1.810, all below the conservative threshold of 3.3. These results suggest that common method bias was unlikely to be a serious threat to the interpretation of the findings.

### 4.5 Data analysis

The data analysis proceeded in four steps. First, descriptive statistics were calculated to examine the distribution of the main variables. Second, confirmatory factor analysis was conducted to assess the reliability, convergent validity, and discriminant validity of the measurement model. Composite reliability, average variance extracted, and the Fornell-Larcker criterion were used to evaluate convergent and discriminant validity (Fornell and Larcker, 1981; Hair et al., 2022), and the heterotrait-monotrait ratio was used as an additional test of discriminant validity (Henseler et al., 2015). Third, structural equation modeling was used to examine the hypothesized associations among perceived threat severity, perceived threat vulnerability, response efficacy, self-efficacy, AI anxiety, protection motivation, and AI learning intention. Bootstrapping was used to estimate indirect associations and confidence intervals. Fourth, fsQCA was used as a complementary configurational method to identify different combinations of conditions associated with high AI learning intention (Ragin, 2008; Fiss, 2011). SEM was used to estimate average structural associations, whereas fsQCA was used to examine whether multiple configurations of cognitive, emotional, and motivational conditions were linked to high AI learning intention.

A post-hoc statistical power analysis was also conducted to evaluate whether the sample size was sufficient for the main structural relationships. Based on the regression model predicting protection motivation from perceived threat severity, perceived threat vulnerability, AI anxiety, response efficacy, and self-efficacy, the explained variance was *R*^2^ = 0.402. This corresponds to an effect size of *f*^2^ = 0.671. With *N* = 350, five predictors, and alpha = 0.05, the achieved power for detecting the overall regression effect exceeded 0.99. Because several mediation paths involved small indirect associations, these results were interpreted cautiously with attention to effect sizes and confidence intervals rather than statistical significance alone.

## 5 Results

### 5.1 Descriptive statistics

The mean scores of all measurement items ranged from 4.246 to 4.634, indicating that the surveyed university students generally exhibited moderately high levels of perceived threat, AI anxiety, protection motivation, learning intention, and efficacy beliefs. Standard deviations ranged from 1.498 to 1.718, suggesting some dispersion in responses while remaining within an acceptable range overall. Item skewness ranged from −0.348 to −0.037 and kurtosis ranged from −0.911 to −0.411, indicating that the distributions were generally stable and showed no obvious skewness or extreme peakedness.

### 5.2 Measurement model

The reliability and convergent validity of the measurement model were assessed using standardized factor loadings, composite reliability, and average variance extracted. As shown in Table 2, all factor loadings exceeded 0.75, the composite reliability values were above 0.85, and the AVE values exceeded 0.60. These results indicate acceptable convergent validity and internal consistency. Consistent with the scale-development logic described above, the 12 AI anxiety items were retained as indicators of a global AI anxiety construct rather than as four independent subscales.

**Table 2:** Reliability and convergent validity.

Construct	Item	Factor loading	CR	AVE
PTS	PTS1	0.816	0.878	0.644
PTS	PTS2	0.791	0.878	0.644
PTS	PTS3	0.794	0.878	0.644
PTS	PTS4	0.808	0.878	0.644
PTV	PTV1	0.780	0.870	0.627
PTV	PTV2	0.800	0.870	0.627
PTV	PTV3	0.786	0.870	0.627
PTV	PTV4	0.800	0.870	0.627
AIA	AIA1	0.781	0.949	0.630
AIA	AIA2	0.780	0.949	0.630
AIA	AIA3	0.781	0.949	0.630
AIA	AIA4	0.768	0.949	0.630
AIA	AIA5	0.790	0.949	0.630
AIA	AIA6	0.815	0.949	0.630
AIA	AIA7	0.792	0.949	0.630
AIA	AIA8	0.812	0.949	0.630
AIA	AIA9	0.801	0.949	0.630
AIA	AIA10	0.803	0.949	0.630
AIA	AIA11	0.808	0.949	0.630
AIA	AIA12	0.785	0.949	0.630
PM	PM1	0.788	0.861	0.608
PM	PM2	0.757	0.861	0.608
PM	PM3	0.787	0.861	0.608
PM	PM4	0.786	0.861	0.608
AIIN	AIIN1	0.775	0.870	0.626
AIIN	AIIN2	0.797	0.870	0.626
AIIN	AIIN3	0.771	0.870	0.626
AIIN	AIIN4	0.821	0.870	0.626
RE	RE1	0.779	0.858	0.602
RE	RE2	0.780	0.858	0.602
RE	RE3	0.761	0.858	0.602
RE	RE4	0.784	0.858	0.602
SE	SE1	0.784	0.877	0.640
SE	SE2	0.810	0.877	0.640
SE	SE3	0.818	0.877	0.640
SE	SE4	0.787	0.877	0.640

Discriminant validity was assessed using both the Fornell-Larcker criterion and the heterotrait-monotrait ratio of correlations. As shown in Table 3, the square root of each construct’s AVE was greater than its correlations with other constructs. In addition, all HTMT values were below 0.85, with the largest value being 0.632 (Table 4). These results provide support for discriminant validity among the seven constructs (see Table 5).

**Table 3:** Discriminant validity using the Fornell-Larcker criterion.

Construct	SE	AIA	RE	AIIN	PM	PTV	PTS
SE	0.800						
AIA	0.471	0.794					
RE	0.451	0.387	0.776				
AIIN	0.488	0.441	0.447	0.791			
PM	0.468	0.428	0.382	0.465	0.780		
PTV	0.470	0.529	0.449	0.493	0.479	0.792	
PTS	0.444	0.409	0.388	0.422	0.544	0.489	0.802
AVE	0.640	0.630	0.602	0.626	0.608	0.627	0.644

**Table 4:** HTMT results.

Construct	SE	AIA	RE	AIIN	PM	PTV	PTS
SE	—						
AIA	0.517	—					
RE	0.523	0.432	—				
AIIN	0.563	0.491	0.526	—			
PM	0.538	0.475	0.447	0.543	—		
PTV	0.537	0.584	0.522	0.571	0.553	—	
PTS	0.510	0.453	0.454	0.493	0.632	0.564	—

**Table 5:** VIF diagnostics.

Predictor	VIF in the PM equation
Perceived threat severity	1.475
Perceived threat vulnerability	1.721
AI anxiety	1.565
Response efficacy	1.423
Self-efficacy	1.572

### 5.3 Structural model and hypothesis testing

The structural equation model showed good fit. As shown in Table 6, the model had χ^2^/df = 1.629, RMSEA = 0.042, TLI = 0.948, CFI = 0.952, and IFI = 0.952. These indices indicate that the structural model was acceptable for hypothesis testing.

**Table 6:** Model fit indices.

Model fit	Criteria	Model fit of research model
MLχ^2^	Smaller is better	954.778
DF	Larger is better	586
Normed Chi-sqr (χ^2^/DF)	1 < χ^2^/DF < 3	1.629
RMSEA	< 0.08	0.042
TLI (NNFI)	> 0.90	0.948
CFI	> 0.90	0.952
IFI	> 0.90	0.952

The path results are shown in Table 7. Perceived threat severity and perceived threat vulnerability were positively associated with AI anxiety and protection motivation. Self-efficacy and AI anxiety were positively associated with protection motivation. Response efficacy showed a positive but statistically non-significant association with protection motivation. Protection motivation was positively associated with AI learning intention. Therefore, H1–H4 and H6–H8 were supported, whereas H5 was not supported.

**Table 7:** Structural path results.

Path	Standardized coefficient	Standard error	T value	Significance
PTS → AIA	0.198	0.051	3.876	***
PTV → AIA	0.432	0.051	8.447	***
PTS → PM	0.322	0.051	6.358	***
PTV → PM	0.154	0.055	2.811	0.005
RE → PM	0.069	0.050	1.385	0.167
SE → PM	0.172	0.052	3.281	0.001
AIA → PM	0.107	0.052	2.054	0.041
PM → AIIN	0.465	0.047	9.807	***

### 5.4 Indirect associations

Bootstrapping was used to test indirect associations. As shown in Table 8, the indirect associations from perceived threat severity and perceived threat vulnerability to protection motivation through AI anxiety were statistically significant but small. The indirect associations from perceived threat severity, AI anxiety, perceived threat vulnerability, and self-efficacy to AI learning intention through protection motivation were statistically significant. The indirect association from response efficacy to AI learning intention through protection motivation was not statistically significant. Therefore, H9, H10, H11, H12, H13, and H15 were supported, whereas H14 was not supported.

**Table 8:** Indirect associations.

Mediation path	Point estimate	Lower bound	Upper bound	*p*-value
PTS → AIA →PM	0.021	0.001	0.048	0.042
PTV →AIA →PM	0.046	0.002	0.096	0.041
PTS →PM →AIIN	0.150	0.101	0.204	0.000
AIA →PM →AIIN	0.050	0.002	0.102	0.041
PTV →PM →AIIN	0.072	0.017	0.134	0.011
RE →PM →AIIN	0.032	−0.011	0.080	0.147
SE →PM →AIIN	0.080	0.032	0.133	0.001

### 5.5 fsQCA configurational analysis

Structural equation modeling estimates average net associations among variables, but AI learning intention may also be associated with different combinations of psychological conditions. Therefore, this study further employed fuzzy-set qualitative comparative analysis to examine configurational patterns associated with high AI learning intention. Perceived threat severity, perceived threat vulnerability, response efficacy, self-efficacy, AI anxiety, and protection motivation were treated as causal conditions. AI learning intention was treated as the outcome.

This study used the direct-calibration method recommended in fsQCA research (Ragin, 2008; Fiss, 2011). Because all variables were measured on a seven-point Likert scale, three theoretically interpretable anchors were selected: 6 as the threshold for full membership, 4 as the crossover point, and 2 as the threshold for full non-membership. On the questionnaire scale, 6 indicates a relatively high level of agreement, 4 represents a neutral position, and 2 indicates a relatively low level of agreement. This calibration strategy is appropriate because the constructs reflect psychological agreement rather than objective external thresholds (see Table 9).

**Table 9:** Calibration anchors for fsQCA variables.

Variable	Abbreviation	Full membership	Crossover point	Full non-membership
Perceived threat severity	PTS	6	4	2
Perceived threat vulnerability	PTV	6	4	2
Response efficacy	RE	6	4	2
Self-efficacy	SE	6	4	2
AI anxiety	AIA	6	4	2
Protection motivation	PM	6	4	2
AI learning intention	AIIN	6	4	2

Necessary-condition analysis was first conducted to examine whether any single condition was necessary for high AI learning intention. A condition is generally considered necessary only when its consistency is greater than 0.90. As shown in Table 10, none of the single conditions reached the 0.90 threshold. This suggests that high AI learning intention among Chinese university students is not dependent on any single psychological factor.

**Table 10:** Necessary-condition analysis for high AI learning intention.

Condition	Consistency	Coverage
SE	0.821	0.779
PM	0.800	0.782
RE	0.794	0.779
AIA	0.785	0.783
PTV	0.783	0.794
PTS	0.783	0.783
~AIA	0.453	0.656
~PTS	0.438	0.632
~PTV	0.436	0.617
~RE	0.427	0.633
~PM	0.409	0.611
~SE	0.394	0.617

A truth table was constructed using six causal conditions. Given the sample size of 350 and the use of six causal conditions, the frequency threshold was set at 3 cases. The consistency threshold was set at 0.85, and the PRI consistency threshold was set at 0.75. The retained truth-table rows are reported in Supplementary Table S2. Both parsimonious and intermediate solutions were examined. Conditions appearing in both solutions were treated as core conditions, whereas conditions appearing only in the intermediate solution were treated as peripheral conditions (see Table 11).

**Table 11:** Intermediate fsQCA solution for high AI learning intention.

Condition	S1: Efficacy-motivation	S2: Threat-motivation	S3: Anxiety-efficacy
PTS		●	
PTV		●	
RE	●		●
SE	•		
AIA			•
PM	●	●	●
Raw coverage	0.605	0.614	0.594
Unique coverage	0.024	0.074	0.019
Consistency	0.881	0.890	0.899
Solution coverage	0.730		
Solution consistency	0.856		

● indicates the presence of a core condition; • indicates the presence of a peripheral condition; blank cells indicate that the condition is not central to the configuration.

The intermediate solution can be expressed as follows: High AIIN = RE * SE * PM + PTS * PTV * PM + RE * AIA * PM. The first configuration can be described as an efficacy-motivation configuration. In this pathway, response efficacy and protection motivation serve as core conditions, while self-efficacy functions as a peripheral condition. The second configuration can be described as a threat-motivation configuration. In this pathway, perceived threat severity, perceived threat vulnerability, and protection motivation all appear as core conditions. The third configuration can be described as an anxiety-efficacy configuration. In this pathway, response efficacy and protection motivation appear as core conditions, while AI anxiety appears as a peripheral condition. This result does not imply that AI anxiety is automatically transformed into learning intention. Rather, it suggests that AI anxiety is associated with high AI learning intention only when it appears together with the belief that learning AI is useful and with strong protection motivation.

## 6 Discussion

### 6.1 Summary of main findings

This study examined AI learning intention among Chinese university students from a Protection Motivation Theory perspective. Using SEM and fsQCA as complementary analytical approaches, the study found that perceived threat severity and perceived threat vulnerability were positively associated with both AI anxiety and protection motivation. Self-efficacy and AI anxiety were positively associated with protection motivation, whereas response efficacy showed a positive but statistically non-significant direct association with protection motivation in the SEM model. Protection motivation was positively associated with AI learning intention and served as the most proximal motivational construct in the model. The mediation results showed that AI anxiety had small but statistically significant indirect associations between threat appraisal and protection motivation. The fsQCA results further indicated that high AI learning intention was associated with multiple configurations rather than with a single psychological condition.

These findings should be interpreted in line with the cross-sectional and self-reported nature of the data. The study does not claim to demonstrate temporal change or causal transformation from AI anxiety to AI learning intention. Instead, it provides evidence of structural associations, indirect relationships, and configurational patterns among threat appraisal, efficacy beliefs, AI anxiety, protection motivation, and AI learning intention.

### 6.2 Response efficacy: non-significant net effect but important configurational role

One of the most important findings concerns the role of response efficacy. In the SEM model, response efficacy had a positive but statistically non-significant association with protection motivation. At first glance, this appears inconsistent with many traditional PMT studies, especially in health-protection and risk-prevention contexts, where response efficacy often serves as a strong predictor of protective behavior. In those contexts, the protective response is usually concrete and its expected outcome is relatively clear. For example, individuals may believe that vaccination reduces disease risk or that safety behavior reduces exposure to harm. In such cases, belief in the effectiveness of the response is closely tied to behavioral motivation.

AI learning differs from these traditional PMT contexts. For university students, the belief that learning AI is useful may be widely accepted but still too general to directly produce strong motivation. AI learning is not a single protective act with an immediate and visible outcome. It is a long-term capability-building process involving uncertain learning pathways, changing tools, different disciplinary needs, and unclear labor-market returns. Therefore, response efficacy may function more as a background belief than as a direct motivational trigger. Students may agree that learning AI is useful, but this belief alone may not be sufficient to generate protection motivation unless they also feel capable of learning AI or perceive a clear need to act.

This interpretation is supported by the contrast between the SEM and fsQCA results. Although response efficacy was not a significant independent predictor in the SEM model, it appeared as a core condition in two fsQCA configurations: the efficacy-motivation configuration and the anxiety-efficacy configuration. This suggests that response efficacy should not be interpreted as irrelevant. Rather, its role appears to be conditional and configurational. It becomes important when it is combined with protection motivation and either self-efficacy or AI anxiety.

Theoretically, this result refines the application of PMT in AI learning contexts. In traditional risk settings, response efficacy often answers whether a protective action works. In AI learning settings, this belief may need to be accompanied by perceived capability, emotional salience, or clear protection motivation before it becomes behaviorally meaningful. Thus, the study suggests that response efficacy in AI learning may be better understood as a conditional contributor rather than as a stable independent predictor.

### 6.3 The small indirect role of AI anxiety

The mediation results showed that AI anxiety had statistically significant indirect associations between perceived threat severity and protection motivation and between perceived threat vulnerability and protection motivation. However, the magnitude of these indirect associations was small. Therefore, AI anxiety should not be described as a dominant mechanism. A more cautious interpretation is that AI anxiety provides a modest emotional link between threat appraisal and protection motivation.

This finding is theoretically meaningful precisely because the effect is small. It suggests that threat appraisal is not connected to protection motivation only through emotional anxiety. Students may form protection motivation directly from cognitive appraisals of future risk, employment pressure, or skill renewal demands. AI anxiety adds an emotional layer to this process, but it does not fully explain it. In other words, anxiety may help make AI-related threats psychologically salient, but it is not sufficient by itself to account for students’ motivation to learn AI.

The fsQCA results further suggest that AI anxiety appears in a high-learning-intention configuration only when it is combined with response efficacy and protection motivation. This means that anxiety is more likely to be associated with adaptive learning intention when students also believe that AI learning is useful and when they possess a clear motivational orientation toward capability improvement. Practically, this finding suggests that universities should not deliberately increase students’ anxiety in order to motivate AI learning. Educational interventions should instead help students understand AI-related changes realistically while strengthening their self-efficacy, response efficacy, and access to structured AI learning opportunities.

### 6.4 Chinese higher education context

The Chinese higher education context provides an important background for interpreting the associations among perceived threat vulnerability, AI anxiety, protection motivation, and AI learning intention. Chinese university students face strong employment competition, performance-oriented educational expectations, and increasing pressure to develop practical competencies before graduation. In this environment, AI is not perceived only as a technological tool. It is also understood as a force that may influence employability, major-related skill value, and future occupational mobility.

This context helps explain why perceived threat vulnerability is closely related to AI anxiety and protection motivation. Perceived threat vulnerability reflects students’ judgments that their own learning, major, or future employment may be affected by AI. For students preparing to enter a competitive labor market, this sense of personal relevance may be more psychologically salient than general awareness that AI is important. When students believe that AI may affect their own major, career direction, or competitiveness relative to peers, AI-related pressure becomes more immediate. This helps explain why perceived vulnerability is strongly associated with AI anxiety.

At the same time, perceived vulnerability is also associated with protection motivation. In a performance-oriented higher education environment, students are often encouraged to respond to uncertainty through skill acquisition, credential accumulation, and self-improvement. Therefore, AI-related threat perception may be linked not only to worry, but also to a stronger perceived need to improve AI-related competencies. Protection motivation, in this sense, reflects students’ readiness to respond to AI-related uncertainty through learning rather than avoidance.

The measurement items in this study also reflect this contextual logic. AI anxiety was not measured merely as discomfort with machines or unfamiliar technology. It included concerns about future employment, difficulty keeping up with rapid AI development, pressure from peers who use AI tools, insufficient AI knowledge and skills, possible weakening of existing professional advantages, and instability in future career planning. These concerns are closely connected to the lived educational and employment pressures faced by university students. Therefore, AI anxiety in this study should be interpreted as a form of learning- and career-related technological anxiety rather than as a general fear of technology.

### 6.5 Theoretical and practical implications

This study makes three theoretical contributions. First, it extends PMT in the context of AI learning intention while avoiding the assumption that AI learning is equivalent to general technology adoption. AI learning intention refers to students’ willingness to invest in future-oriented AI competencies, not simply their intention to use a particular AI tool. Second, the study contributes to the literature on AI anxiety by offering a cautious account of its role. AI anxiety is neither treated as purely harmful nor described as automatically beneficial. Third, the study shows the value of distinguishing between net-effect and configurational explanations. SEM results indicate that self-efficacy and AI anxiety are more stable predictors of protection motivation than response efficacy, while fsQCA results show that response efficacy still matters in specific configurations.

The findings also have practical implications for universities. AI education should not rely only on emphasizing the usefulness of AI. Because response efficacy alone was not a significant independent predictor in the SEM model, simply telling students that AI is useful may not be enough. Universities should combine usefulness-oriented messages with hands-on learning opportunities that strengthen students’ confidence in their ability to learn AI. In addition, AI anxiety should be addressed through structured support rather than fear-based motivation. Career education and AI education should also be better integrated so that students can understand how AI is changing different fields, what level of AI competence is expected in different career pathways, and how students from different majors can develop realistic AI learning plans.

### 6.6 Limitations and future research

Several limitations should be acknowledged. First, the study used cross-sectional self-reported survey data. Therefore, the findings should be interpreted as associations rather than causal evidence. Future studies could use longitudinal or experimental designs to examine how AI anxiety, efficacy beliefs, protection motivation, and learning intention change over time.

Second, although the AI anxiety scale showed good reliability and validity in this study, it was developed and contextualized for Chinese university students and was modeled as a global latent construct. Future studies should further validate the scale across different student groups, institutional settings, disciplines, and cultural contexts, and should compare alternative measurement structures, such as first-order multidimensional and second-order factor models. Third, the mediation effects involving AI anxiety were small. Future studies should examine additional emotional and motivational mechanisms, such as perceived control, future career anxiety, learning engagement, academic resilience, and AI literacy. Fourth, fsQCA identified several configurations associated with high AI learning intention, but the stability of these configurations should be further tested with larger and more diverse samples.

## 7 Conclusion

This study examined AI anxiety and AI learning intention among Chinese university students from a Protection Motivation Theory perspective. The findings show that perceived threat severity, perceived threat vulnerability, self-efficacy, AI anxiety, and protection motivation are positively associated within the proposed model, while response efficacy plays a more conditional role. Protection motivation is the most proximal construct associated with AI learning intention. AI anxiety provides a small but statistically significant emotional link between threat appraisal and protection motivation, but it should not be interpreted as a dominant mechanism. The fsQCA results further indicate that high AI learning intention is associated with multiple configurations, including efficacy-motivation, threat-motivation, and anxiety-efficacy configurations. Overall, the study suggests that AI learning intention is better understood as the result of jointly operating cognitive, emotional, and motivational conditions rather than as the outcome of a single factor.

## Data Availability

The raw data supporting the conclusions of this article will be made available by the authors, without undue reservation.
