# Supplementary material for: AI anxiety and AI learning intention among Chinese university students: a protection motivation theory perspective
Source: Front Psychol. 2026 Jul 10;17:1865077. doi: 10.3389/fpsyg.2026.1865077 (PMC13395771; doi:10.3389/fpsyg.2026.1865077)
Supplement: Supplementary file 1 [file Supplementary_file_1.DOCX]

**Supplementary Material**

*Supplementary Material for: AI Anxiety and AI Learning Intention among Chinese University Students: A Protection Motivation Theory Perspective*

S1. Measurement Items

All items were measured using a seven-point Likert scale, ranging from 1 = strongly disagree to 7 = strongly agree.

**Supplementary Table S1. Measurement items**

| **Construct** | **Code** | **Item** |
| --- | --- | --- |
| Perceived Threat Severity | PTS1 | The development of artificial intelligence may significantly change the future employment environment of university students. |
| Perceived Threat Severity | PTS2 | If university students cannot adapt to the development of artificial intelligence, their future career competitiveness may be significantly affected. |
| Perceived Threat Severity | PTS3 | Artificial intelligence may reduce the value of some traditional knowledge and skills. |
| Perceived Threat Severity | PTS4 | The pressure of capability renewal brought about by artificial intelligence has a considerable influence on the future development of university students. |
| Perceived Threat Vulnerability | PTV1 | I think my future learning or employment may be affected by the development of artificial intelligence. |
| Perceived Threat Vulnerability | PTV2 | If I do not learn AI-related knowledge, I may be at a disadvantage in future competition. |
| Perceived Threat Vulnerability | PTV3 | My major or future career direction may be affected by artificial intelligence technologies. |
| Perceived Threat Vulnerability | PTV4 | Compared with classmates who have already mastered AI skills, I may be more likely to feel competitive pressure. |
| Response Efficacy | RE1 | Learning artificial intelligence knowledge can help me better cope with future learning and employment pressure. |
| Response Efficacy | RE2 | Mastering artificial intelligence tools can improve my learning efficiency and problem-solving ability. |
| Response Efficacy | RE3 | Learning AI-related skills can enhance my career competitiveness. |
| Response Efficacy | RE4 | Actively learning artificial intelligence can help reduce my uncertainty when facing technological change. |
| Self-Efficacy | SE1 | I believe that I can understand basic knowledge related to artificial intelligence. |
| Self-Efficacy | SE2 | I believe that I can learn to use common artificial intelligence tools. |
| Self-Efficacy | SE3 | Even if AI knowledge is somewhat difficult, I am confident that I can gradually master it through effort. |
| Self-Efficacy | SE4 | When I encounter problems in AI learning, I am able to find materials or methods to solve them. |
| AI Anxiety | AIA1 | When I think that artificial intelligence may affect future employment, I feel worried. |
| AI Anxiety | AIA2 | The development of artificial intelligence is so fast that I worry I may not be able to keep up in time. |
| AI Anxiety | AIA3 | When I see classmates skillfully using AI tools, I sometimes feel pressure. |
| AI Anxiety | AIA4 | I worry that I do not have sufficient AI knowledge and skills. |
| AI Anxiety | AIA5 | When I need to learn new AI tools or AI courses, I sometimes feel nervous. |
| AI Anxiety | AIA6 | I worry that many future jobs will prefer people who are familiar with AI technologies. |
| AI Anxiety | AIA7 | I worry that artificial intelligence will weaken the advantages of my existing professional knowledge. |
| AI Anxiety | AIA8 | When facing AI-generated content or results, I sometimes feel uncertain or uneasy. |
| AI Anxiety | AIA9 | I worry that excessive reliance on AI may affect my independent thinking ability. |
| AI Anxiety | AIA10 | I worry that changes brought about by artificial intelligence will make future career planning more unstable. |
| AI Anxiety | AIA11 | When discussing AI replacing human work, I feel a certain degree of unease. |
| AI Anxiety | AIA12 | Overall, the development of artificial intelligence makes me feel a certain degree of learning and development pressure. |
| Protection Motivation | PM1 | Facing the development of artificial intelligence, I think it is necessary for me to actively improve relevant abilities. |
| Protection Motivation | PM2 | To cope with future uncertainty, I have a strong motivation to learn AI-related knowledge. |
| Protection Motivation | PM3 | Learning artificial intelligence is an important form of self-preparation for me. |
| Protection Motivation | PM4 | I am willing to improve my AI abilities to enhance my adaptability to future learning and employment changes. |
| AI Learning Intention | AIIN1 | In the future, I am willing to actively learn AI-related knowledge. |
| AI Learning Intention | AIIN2 | I am willing to invest time in practicing and mastering common AI tools. |
| AI Learning Intention | AIIN3 | If there is an opportunity, I am willing to participate in AI-related courses, lectures, or training. |
| AI Learning Intention | AIIN4 | I plan to continue paying attention to the application of artificial intelligence in learning, my major, and employment. |

S2. Supplementary fsQCA Tables

**Supplementary Table S2. Retained truth-table rows for high AI learning intention**

| **PTS** | **PTV** | **RE** | **SE** | **AIA** | **PM** | **Frequency** | **Consistency** | **PRI consistency** | **Raw coverage** |
| --- | --- | --- | --- | --- | --- | --- | --- | --- | --- |
| 1 | 1 | 1 | 1 | 1 | 1 | 84 | 0.932 | 0.882 | 0.469 |
| 1 | 1 | 1 | 1 | 0 | 1 | 16 | 0.940 | 0.831 | 0.260 |
| 0 | 1 | 1 | 1 | 1 | 1 | 15 | 0.931 | 0.813 | 0.242 |
| 1 | 1 | 0 | 1 | 1 | 1 | 14 | 0.936 | 0.826 | 0.252 |
| 1 | 1 | 1 | 0 | 1 | 1 | 11 | 0.943 | 0.818 | 0.228 |
| 1 | 1 | 0 | 1 | 0 | 1 | 8 | 0.930 | 0.753 | 0.196 |

Note. Frequency threshold = 3; consistency threshold = 0.85; PRI consistency threshold = 0.75.

**Supplementary Table S3. Parsimonious solution for high AI learning intention**

| **Configuration** | **Expression** | **Raw coverage** | **Unique coverage** | **Consistency** |
| --- | --- | --- | --- | --- |
| P1 | RE * PM | 0.677 | 0.132 | 0.861 |
| P2 | PTS * PTV * PM | 0.614 | 0.068 | 0.890 |
| Solution coverage |  | 0.746 |  |  |
| Solution consistency |  | 0.847 |  |  |
